# Cardiac profile of the Czech population of Duchenne muscular dystrophy patients: a cardiovascular magnetic resonance study with T1 mapping

**DOI:** 10.1186/s13023-018-0986-0

**Published:** 2019-01-09

**Authors:** Roman Panovský, Martin Pešl, Tomáš Holeček, Jan Máchal, Věra Feitová, Lenka Mrázová, Jana Haberlová, Alžběta Slabá, Pavel Vít, Veronika Stará, Vladimír Kincl

**Affiliations:** 1 International Clinical Research Center, St. Anne’s University Hospital, Brno, Czech Republic; 2 1st Department of Internal Medicine/Cardioangiology, St. Anne’s University Hospital, Faculty of Medicine, Masaryk University, Brno, Czech Republic; 3 Department of Biology, Faculty of Medicine, Masaryk University, Brno, Czech Republic; 4 Department of Medical Imaging, St. Anne’s University Hospital, Brno, Czech Republic; 5 Department of Pathophysiology, Faculty of Medicine, Masaryk University, Brno, Czech Republic; 6 Department of Pediatric Neurology, University Hospital Brno, Brno, Czech Republic; 7 Department of Pediatric Neurology, University Hospital Motol, Second Faculty of Medicine, Charles University, Prague, Czech Republic; 8 Pediatric Clinic, University Hospital Brno, Brno, Czech Republic; 9 Department of Pediatrics, University Hospital Motol, Second Faculty of Medicine, Charles University, Prague, Czech Republic

**Keywords:** Cardiac magnetic resonance, Duchene muscular dystrophy, T1 mapping; extracellular volume, Cardiomyopathy

## Abstract

**Background:**

The progressive cardiomyopathy that develops in boys with Duchenne and Becker muscular dystrophy (DMD/BMD) is presumed to be a secondary consequence of the fibrosis within the myocardium. There are only limited data on using parametric imaging in these patients. The purpose of this study was to assess native T1 and extracellular volume (ECV) values in DMD patients.

**Methods:**

The Czech population of males with DMD/BMD was screened. All eligible patients fulfilling the inclusion criteria were included. Forty nine males underwent cardiac magnetic resonance (MR) examination including T1 native and post-contrast mapping measurements. One DMD patient and all BMD patients were excluded from statistical analysis. Three groups were compared – Group D1 - DMD patients without late gadolinium enhancement (LGE) (*n* = 23), Group D2 - DMD patients with LGE (*n* = 20), and Group C – gender matched controls (*n* = 13).

**Results:**

Compared to controls, both DMD groups had prolonged T1 native relaxation time. These results are concordant in all 6 segments as well as in global values (1041 ± 31 ms and 1043 ± 37 ms vs. 983 ± 15 ms, both *p* < 0.05). Group D2 had significantly increased global ECV (0.28 ± 0.044 vs. 0.243 ± 0.013, *p* < 0.05) and segmental ECV in inferolateral and anterolateral segments in comparison with controls. The results were also significant after adjustment for subjects’ age.

**Conclusion:**

DMD males had increased native T1 relaxation time independent of the presence or absence of myocardial fibrosis. Cardiac MR may provide clinically useful information even without contrast media administration.

## Background

Muscular dystrophies mostly affect the skeletal muscles, but in the case of dystrophinopathy such as Duchenne and Becker muscular dystrophy (DMD and BMD), the heart muscle can be also seriously affected and the dystrophin deficiency in the heart manifests as a cardiomyopathy. For a long time, the cardiac impairment was significantly under-diagnosed for many reasons, even though patients suffer heart failure and also arrhythmic complications. In last years, the strong progress in cardiac management that includes first of all a regular cardiac assessment as well as early therapeutic recommendations, has been achieved [1, 2].

The progressive cardiomyopathy that develops in male with DMD/BMD is presumed to be a secondary consequence of the fibrosis within the myocardium. As with skeletal muscle, the loss of dystrophin and the disruption of the dystrophin-glycoprotein complex (DGC) cause either fragile sarcolemma of the cardiomyocyte (CM) and muscle contraction damages, leading to small tears in the cell membrane, or stem cell depletion preventing heart muscle regeneration [3, 4].

There are available databases reporting correlation between DMD genotype and phenotype. These include the Leiden muscular dystrophy pages (http://www.dmd.nl) in the Netherlands, and similar databases in Belgium and France [5–7]. Those report ability to distinguish between DMD and BMD of various types of mutations, still cardiac involvement could yet not be predicted.

The degree of cardiomyopathy is not necessarily correlated to the severity of skeletal myopathy and the onset of cardiac disease appears long before the first cardiac symptoms [8]. Due to relatively low physical activity and subsequently low oxygen demand of patients with mobility issues, clinical symptoms of heart failure are rarely pronounced and despite the general recommendations, non-invasive imaging is routinely performed less often than recommended. Moreover, echocardiography, as the most available screening method, is usually severely hampered by skeletal deformities and narrow intercostal spaces. Therefore, heart function is usually very difficult to assess and/or assess reliably [9]. Therefore cardiovascular magnetic resonance (MR) has become a non-invasive diagnostic tool of choice for DMD patients. It is a precise and highly reproducible technique to assess left and right ventricular volumes, masses, and function. Late gadolinium enhancement (LGE) is able to image regional myocardial fibrosis, precedes a decrease of left ventricular (LV) EF, and predicts adverse cardiac events in DMD patients [10–18]. Techniques of parametric mapping, especially T1 mapping, have brought new possibilities for assessing very early cardiac involvement, even before LVEF decline and even before the appearance of LGE [18]. T1 mapping has the potential to assess diffuse slight changes in myocardium. Nevertheless, data on using parametric imaging in patients with DMD/BMD are still limited [19–22].

The purpose of this study was to assess phases of myocardial involvement in DMD/BMD patients using native T1 and extracellular volume (ECV) values. This involvement will be evaluated across DMD mutations of study patients in order to identify patients in higher risk of cardiac fibrosis.

## Methods

### Patient population

The large cohort of the Czech male population with genetically diagnosed DMD/BMD dystrophin mutation (more than 100 patients) was screened by two main Czech neurological centres in cooperation with the Czech Parent Project organization [23, 24]. All eligible patients fulfilling the inclusion criteria were included into the study. The inclusion criteria were: 1/signed informed consent by the patient (or by the patient and his parents in the case of a child); 2/absence of MR contraindications such as an implanted pacemaker/defibrillator, cochlear implant, other ferromagnetic metal parts in the patient’s body, claustrophobia, etc.; 3/absence of contraindication for using contrast media such as severe renal insufficiency; 4/patient’s ability to co-operate during MR examination; 5/no known cardiovascular pathology apart from dystrophin cardiomyopathies. Finally, from the total 49 DMD/BMD examined males, while 43 DMD patients were included into the analysis, on the other hand, due to differing neurological progression patterns, 5 BMD boys were not included for further analysis. One DMD patient was also excluded from the analysis due to inability to acquire post contrast images - patient had only pre-contrast cardiac MR because of a problem with the intravenous line introduction. Thirteen boys without with clinical indication for cardiac MR by attending paediatricians with cardiology specialization as search for aetiology of unexplained syncope and palpitations without verified rhythm disturbances served as a control group. The basic characteristics of both groups are shown in Table 1.

**Table 1:** Basic characteristic of the study groups

	Group C (Controls) total *n* = 13	Group D (DMD) total *n* = 43
Age [years]	16.5 ± 2.4*	13.8 ± 4.6
Weight [kg]	67.5 ± 11.9*	47.6 ± 19.9
Height [cm]	176 ± 9*	147 ± 19
Dyspnoea [*n* (%)]	0 (0%)	6 (13.6%)
Hypertension [*n* (%)]	0 (0%)	3 (6.8%)
Diabetes [*n* (%)]	1 (7.7%)	0 (0%)
Corticosteroids [*n* (%)]	0 (0%)*	21 (47.7%)
ACE-inhibitors [*n* (%)]	0 (0%)*	18 (40.9%)
ARBs [*n* (%)]	0 (0%)	2 (4.5%)
β-blockers [*n* (%)]	0 (0%)	8 (18.2%)
Diuretics [*n* (%)]	0 (0%)	3 (6.8%)
Stage ambulatory/non-ambulatory [*n* (%)]	–	16 (37.2%) / 27 (62.8%)

Continuous variables are expressed as the mean ± standard deviation, binary variables as count (percentage), and ordinal variables as median (lower – upper quartile)

*DMD* Duchenne muscular dystrophy, *BMI* body mass index (kg/m^2^), *ACE* angiotensin converting enzyme, *ARB* angiotensin receptor blockers; * = *P*-values < 0.05

The study was performed in accordance with the Declaration of Helsinki (2000) of the World Medical Association, and was approved by the institutional ethics committee (University Hospital Brno, reference number 20130410–03). Written informed consent was obtained from the subjects and/or their legally authorized representative.

### Cardiac MR data acquisition

MR studies were performed according to the standard protocol using 1.5 T scanners (Ingenia, Philips Medical Systems, Best, The Netherlands) equipped with 5- and 32-element phased array receiver coils allowing for the use of parallel acquisition techniques in the supine position in repeated breath-hold. Functional imaging using balanced steady state free precession (SSFP, b-TFE) cine sequences included four-chamber, two-chamber and LVOT (left ventricular outflow track) long axis views, and a short axis (SAX) stack from the cardiac base to the apex in the perpendicular plane to the LV long axis. Wall motion abnormalities were assessed. LV functional and morphological parameters were calculated from the SAX stack using the summation-of-disc methods in accordance with the recommendations on post-processing evaluation from the SCMR (Society for Cardiovascular Magnetic Resonance) [25].

LGE images in all long-axis views and the SAX view were acquired 10 min after an intravenous bolus of 0.2 mmol/kg of the gadolinium-based contrast agent gadobutrol (Gadovist, Bayer-Schering Pharma, Germany) using a contrast-sensitive segmented rephased turbo field sequence with slice selective inversion recovery technique (inversion-recovery turbo field echo- IR-TFE) and, in case of doubt, also by phase-sensitive inversion recovery (PSIR) TFE. Both 2-dimensional and 3-diamensional data acquisitions were performed in mid-systole. LGE was defined as an area of visually identified contrast enhancement higher than the mean signal intensity of an adjacent area of the reference myocardium.

Measurements of T1 relaxation times were performed using a modified Look-Locker inversion recovery sequence (MOLLI - balanced single-shot T1-TFE sequence with inversion prepulse, cardiac triggering and breath-hold technique) in the mid-ventricular level in the short-axis plane before and 15 min after contrast agent administration. A 3 s (3 s) 5 s MOLLI scheme for native T1 and 4 s (1 s) 3 s (1 s) 2 s for enhanced T1 mapping was used with typical imaging parameters as follows: FOV 300 × 300 mm, reconstruction matrix 256, slice thickness 10 mm, acquisition voxel size 2.00 × 2.00 × 10.00 mm, time to repetition (TR) ≈ 2.2 ms, echo time (TE) ≈ 1.1 ms, flip angle 35°, SENSE factor 2.

### MR data analysis

T1 native, T1 enhanced and ECV maps were constructed on a pixel-by-pixel basis by using dedicated analysis software cvi42 (Circle Cardiovascular Imaging, Calgary, Canada). Manual epi-and endocardial contours were drawn using 10% borders cutting and a motion correction algorithm was integrated in the analysis. Global and segmental ECV of all six LV segments (segments 7–12 of the American Heart Association 17 segments LV model) [26] were calculated according to the established formula from native and enhanced T1 times, and a haematocrit that was obtained on the same day.

For the detailed analysis, patients with and without LGE were divided into groups based on the presence or absence of LGE as an established marker. Three groups were compared – 1/DMD patients without LGE (Group D1), 2/DMD patients with LGE (Group D2), and 3/controls (Group C). Further, the ability of native cardiac MR data to predict LGE was tested, as well as possible association of medication and motor abilities with native T1 results.

To assess interobserver and intraobserver agreement, 10 native and post-contrast T1 maps were blindly evaluated by two experienced observers (T.H and R.P.), one of them performed the analysis twice.

### Genetic data

Patients provided results of DMD mutation analyses of each individual. Those were available from clinical screening using one or more of the following methods: PCR (polymerase chain reaction), southern blots, DMD gene sequencing and/or genomic hybridisation array, depending on the technology that was available at the time of diagnosis. In two patients the mutation analyses were not disclosed and thus as not available for evaluation. In further 8 patients were present “small” mostly point mutations. Those were not included in the analyses as comparison with exon deletions is complex and often even not comparable at all. As vast majority of mutations were individual ones, groups were predefined. When setting 7 groups, according to hot-spots as published previously [27], four groups had just 2–3 values. Thus the definition of four groups was preferred as follows: Group A represented exon deletion between 2-20th exon (*n* = 6), group B exon deletion between 21-42nd (*n* = 4); C group exon deletion between 43-50th (*n* = 15) and D group of exon deletion between 51st and higher (*n* = 3).

### Statistical analysis

The MR and other continuous data were compared using one-way analysis of variance (ANOVA), followed by the Tukey post hoc test for unequal N in the case of significant effect. Left ventricle volumes, which showed log-normal distribution, underwent logarithmic transformation prior to the analysis. If a significant between-group effect was found, analysis of covariance (ANCOVA) was performed with the selected MR parameter and age as a covariate. The basic characteristics of the DMD patients and the control group were compared by the Kolmogorov-Smirnov test in the case of continuous variables and by Fisher exact test in the case of binary variables. Logistic regression was used for age adjustment in the case of binary data. The logistic regression model using the native MR data was constructed to predict LGE and the area under the ROC (receiver operating characteristics) curve was determined to evaluate the sensitivity and specificity. Student’s t-test for independent data was used to assess the association of either medication or motor abilities with T1 relaxation time.

To assess intraobserver and interobserver agreement in 10 randomly selected subjects, the Friedman test with a Nemenyi post hoc test was employed due to the non-Gaussian distribution of MR parameters in this group. In cases where significant difference was found, Kendall’s W was calculated to assess the level of concordance. Dendrogram with Euclidean distances was used to identify potential clusters of patients based on the cardiac MRI data.

In all cases, results with a *p*-value < 0.05 were considered statistically significant. STATISTICA 13.2 (TIBCO software, USA) was used for the analyses.

## Results

**Fig. 1:**
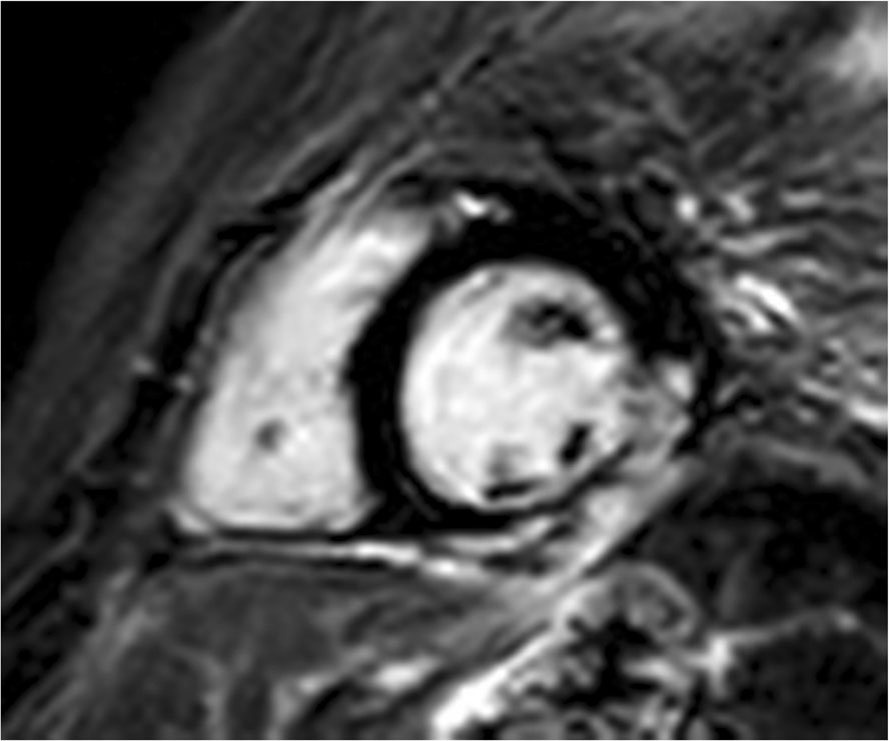
Regional myocardial fibrosis in Duchene Muscular Dystrophy patients – a short axis view with inferolateral late gadolinium enhancement

**Fig. 2:**
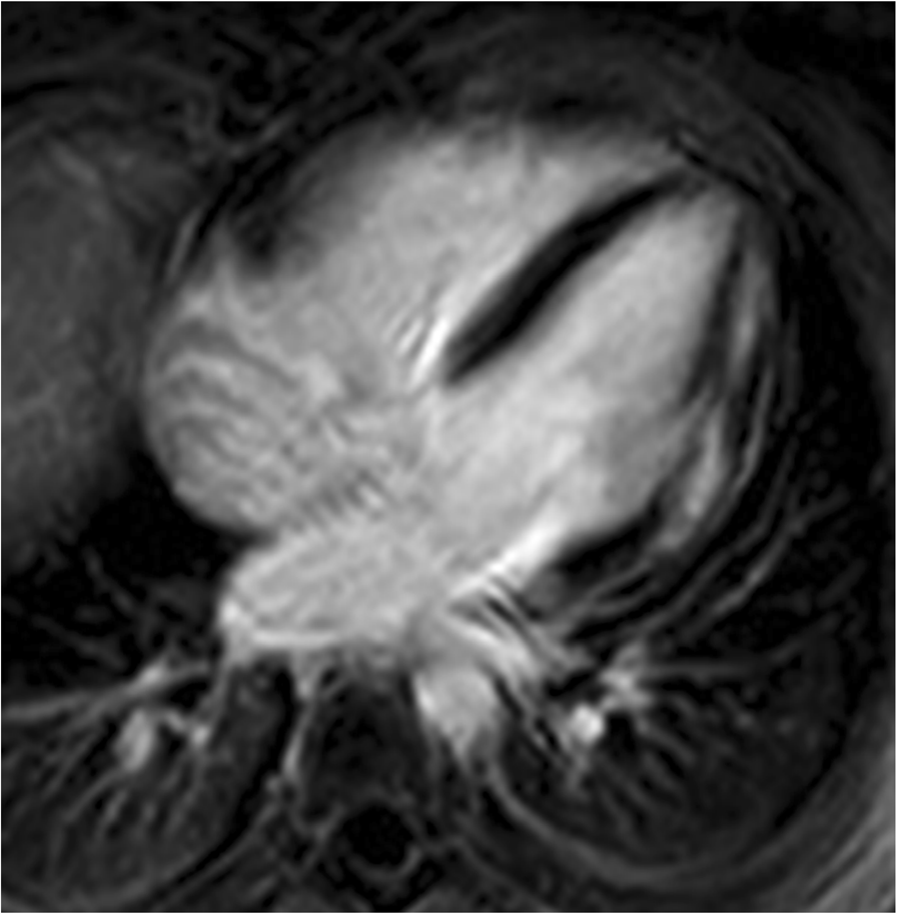
Regional myocardial fibrosis in Duchene Muscular Dystrophy patients – a four-chamber view with anterolateral late gadolinium enhancement

Of the total 44 DMD patients, 1 patient had only pre-contrast cardiac MR because of a problem with the intravenous line introduction. Of 43 patients with post-contrast MR, LGE was found in 20 subjects (47%, Group D2) and 23 boys had no LGE (53%, Group D1). Intramural and subepicardial LGE was found typically in the lateral wall (in all 20 LGE+ subjects) (Figs. 1 and 2) with differing extension to other LV walls – to inferior wall (9 patients) and/or to the interventricular septum and anterior wall (5 patients). No LGE was found in controls (Group C). In comparison to group D1, patients of group D2 were more frequently treated by beta-blockers [7 (35.0%) vs.1 (4.3%); *p* = 0.017] and angiotensin converting enzyme-inhibitors [13 (65.0%) vs. 5 (21.7%); *p* = 0.006]. The patients from D2 group had more often an advanced neurological stage compared to the D1 group - the difference in neurological scale was non-significant after age adjustment (*p* = 0.61).

Table 2 shows a comparison of the selected clinical and MR parameters among the groups. Not surprisingly, compared with other groups, patients of group D1 were younger and had lower weight. Boys from both DMD groups also had lower height. Group D2 had lower LVEF (52.2 ± 14.7 vs. 65.7 ± 7.7 and 65.5% ± 6.9%), one third (7 patients) of group D2 had LVEF< 50% (14% of all DMD patients). Group D1 and D2 had lower stroke volume index (SVi 30 and 33 ml respectively) compared to controls (43 ml). DMD subjects without LGE had a lower LV mass index (LVMi).

**Table 2:** Comparison of selected parameters

	Group C (Controls) *n* = 13	Group D1 (LGE–) *n* = 23	Group D2 (LGE+) *n* = 20	*p* (C vs. D1)		*p* (C vs. D2)		*p* (D1 vs. D2)	
				crude	age-adjusted	crude	age-adjusted	crude	age-adjusted
Age [years]*	16.5 ± 2.4	11.5 ± 2.9	16.5 ± 4.9	**0.003**	**–**	1.00	**–**	**3.10** ^**− 4**^	**–**
Weight [kg]*	67.5 ± 11.9	38.9 ± 12.3	57.7 ± 22.9	**3.10** ^**− 4**^	**2.10** ^**− 4**^	0.31	0.28	**0.003**	**0.002**
Height [cm]*	176 ± 9	139 ± 16	157 ± 18	**1.10** ^**− 4**^	**1.10** ^**− 4**^	**0.006**	**0.006**	**0.002**	**0.002**
LV EF [%]*	65.7 ± 7.7	65.6 ± 7.0	51.9 ± 15.0	1.00	1.00	**0.005**	**0.005**	**5.10** ^**− 4**^	**5.10** ^**− 4**^
**LVMi [g/m** ^**2**^ ]*	51.9 ± 14.2	33.2 ± 7.2	48.0 ± 12.6	**3.10** ^**− 4**^	**2.10** ^**− 4**^	0.64	0.62	**4.10** ^**− 4**^	**3.10** ^**− 4**^
**LV EDVi [ml/m** ^**2**^ **]**	*59 (53–67)*	*50 (38–63)*	*53 (45–74)*	0.23	0.22	0.98	0.98	0.17	0.16
**LV ESVi [ml/m** ^**2**^ ]*	*21 (19–24)*	*17 (13–24)*	*23 (15–49)*	0.60	0.59	0.28	0.27	**0.008**	**0.008**
**LV SVi [ml/m** ^**2**^ ]*	*43 (31–47)*	*32 (26–40)*	*29 (26–34)*	0.10	0.10	**0.016**	**0.017**	0.61	0.61

Variables with the Gaussian distribution are expressed as the mean ± standard deviation; those with different distribution are in *italics* as the *median (lower quartile – upper quartile)*

Variables marked in **bold** and denoted with * have their ANOVA *p*-value < 0.05

*P*-values of the post hoc tests < 0.05 are marked in **bold**

*LGE*– negative late gadolinium enhancement, *LGE+* positive late gadolinium enhancement, *LV* left ventricle, *EF* ejection fraction, *LVMi* left ventricular mass index (g/m^2^), *EDVi* end-diastolic volume index, *ESVi* end-systolic volume indexm, *SVi* stroke volume index

**Fig. 3:**
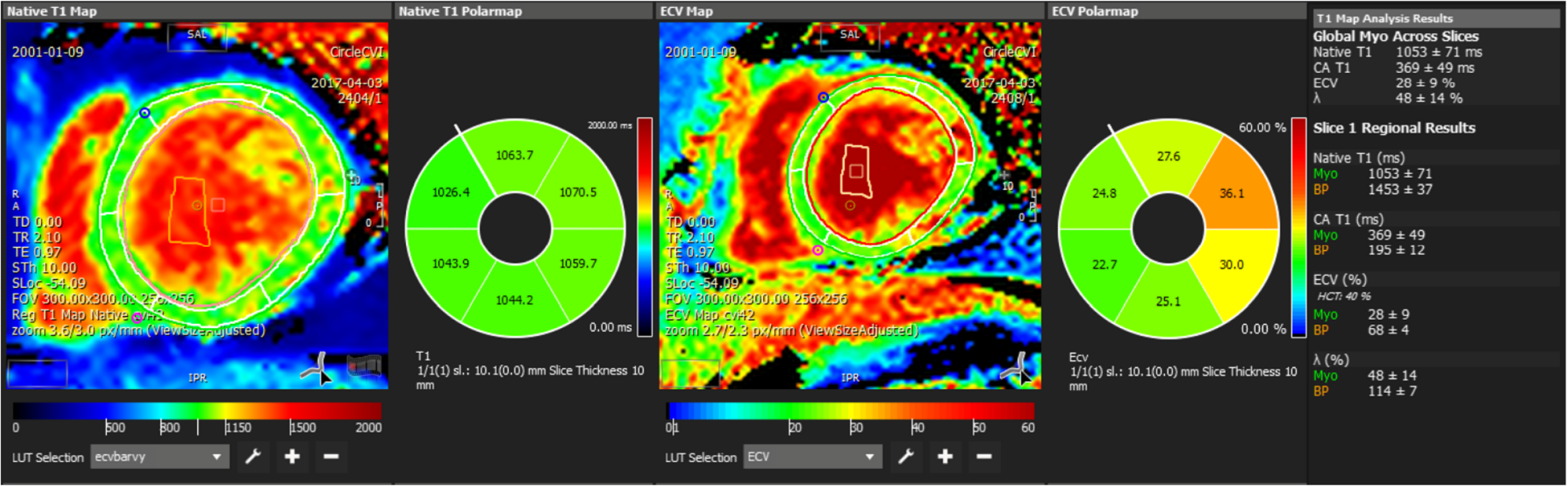
T1 mapping evaluation- native T1 and extracellular volume (ECV) quantification – increased native T1 in all left ventricular segments, increased ECV in anterolateral and inferolateral segments

A comparison of segmental and global native T1 values is shown in Table 3. While there is no difference between groups D1 and D2, both DMD groups had prolonged T1 native relaxation time compared to the control group. These results are concordant in all 6 segments as well as in global values. ECV segmental and global values are listed in Table 4. When compared to the controls, Group D1 did not differ in any segments, but Group D2 had significantly increased global ECV (0.277 ± 0.046 vs. 0.243 ± 0.013) and segmental ECV in inferolateral and anterolateral segments. Both the differences in T1 native relaxation time and in ECV were significant after age adjustment (Fig. 3).

**Table 3:** Comparison of segmental and global native T1 values

Segment	Group C (Controls) *n* = 13	Group D1 (LGE–) *n* = 23	Group D2 (LGE+) *n* = 20	*p* (C vs. D1)		*p* (C vs. D2)		*p* (D1 vs. D2)	
				crude	age-adjusted	crude	age-adjusted	crude	age-adjusted
Anterior*	974 ± 24	1045 ± 51	1046 ± 63	**0.003**	**0.003**	**0.003**	**0.003**	1.00	1.00
Anteroseptal*	992 ± 16	1042 ± 40	1031 ± 26	**5.10** ^**− 4**^	**4.10** ^**− 4**^	**0.006**	**0.005**	0.53	0.51
Inferoseptal*	995 ± 25	1043 ± 40	1035 ± 36	**0.003**	**0.003**	**0.015**	**0.015**	0.73	0.73
Inferior*	978 ± 20	1039 ± 27	1043 ± 55	**6.10** ^**− 4**^	**7.10** ^**− 4**^	**3.10** ^**− 4**^	**4.10** ^**− 4**^	0.95	0.95
Inferolateral*	987 ± 18	1041 ± 39	1059 ± 50	**0.003**	**0.004**	**2.10** ^**− 4**^	**2.10** ^**− 4**^	0.35	0.36
Anterolateral*	965 ± 16	1032 ± 43	1044 ± 62	**0.002**	**0.002**	**3.10** ^**− 4**^	**4.10** ^**− 4**^	0.70	0.70
Global*	983 ± 15	1041 ± 31	1043 ± 37	**2.10** ^**− 4**^	**2.10** ^**− 4**^	**1.10** ^**− 4**^	**1.10** ^**− 4**^	0.96	0.96

Variables are expressed as the mean ± standard deviation

Variables marked in **bold** and denoted with * have their ANOVA *p*-value < 0.05

P-values of the post hoc tests < 0.05 are marked in **bold**

LGE– = negative late gadolinium enhancement; LGE+ = positive late gadolinium enhancement

**Table 4:** Comparison of segmental and global ECV

Segment	Group C (Controls) *n* = 13	Group D1 (LGE–) *n* = 23	Group D2 (LGE+) *n* = 20	*p* (C vs. D1)		*p* (C vs. D2)		*p* (D1 vs. D2)	
				crude	age-adjusted	crude	age-adjusted	crude	age-adjusted
Anterior	0.243 ± 0.017	0.258 ± 0.030	0.266 ± 0.046	0.50	0.50	0.21	0.22	0.76	0.76
Anteroseptal	0.252 ± 0.015	0.258 ± 0.029	0.265 ± 0.057	0.93	0.93	0.69	0.69	0.83	0.83
Inferoseptal	0.251 ± 0.014	0.251 ± 0.030	0.264 ± 0.052	1.00	1.00	0.63	0.62	0.52	0.51
Inferior*	0.237 ± 0.015	0.252 ± 0.029	0.271 ± 0.045	0.50	0.51	**0.033**	**0.035**	0.18	0.18
Inferolateral*	0.236 ± 0.015	0.250 ± 0.031	0.338 ± 0.076	0.75	0.76	**1.10** ^**−4**^	**1.10** ^**− 4**^	**1.10** ^**− 4**^	**1.10** ^**− 4**^
Anterolateral*	0.236 ± 0.016	0.251 ± 0.031	0.296 ± 0.066	0.65	0.65	**0.003**	**0.003**	**0.008**	**0.008**
Global*	0.243 ± 0.013	0.253 ± 0.028	0.280 ± 0.044	0.71	0.71	**0.015**	**0.015**	**0.031**	**0.031**

Variables are expressed as the mean ± standard deviation. Variables marked in **bold** and denoted with * have their ANOVA *p*-value < 0.05

*P*-values of the post hoc tests < 0.05 are marked in **bold**

*ECV* extracellular volume, *LGE–* negative late gadolinium enhancement, *LGE+* positive late gadolinium enhancement

The logistic regression model using the native MR data was constructed to predict LGE, where LV EDVi, LV ESVi and LVMi were identified as significantly contributing factors. The area under ROC (sensitivity-specificity) curve based on all native cardiac MR factors was 0.95 with Youden’s point (point with the highest sum of sensitivity and specificity) corresponding with sensitivity 1.00 and specificity 0.85.

In our study, neither the ACE-inhibitor use nor the motor abilities (ambulatory/non-ambulatory) were associated with the T1 relaxation time. The patients that used beta-blockers had higher global and segmental native T1 values compared with those that did not. This was statistically significant in all segments except of anteroseptal and inferoseptal.

**Fig. 4:**
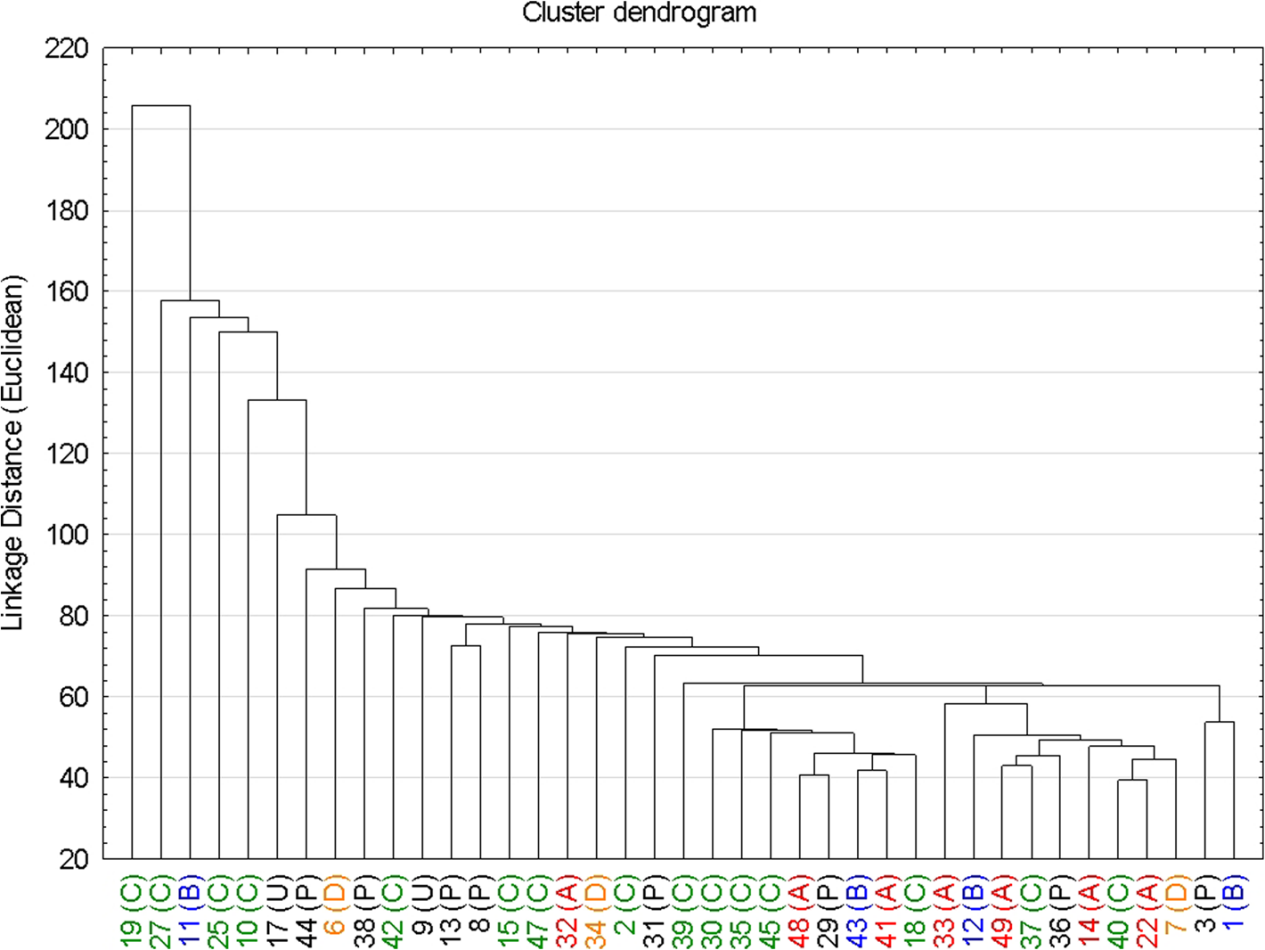
Cluster dendrogram - vertical bars denote the distance between cases and/or their clusters based on the standardized values of cardiac MR parameters - left and right ventricle volumes, segmental T1 native and ECV parameters. Only patients with complete cardiac MR data and genetic analysis are included. A = exon 2–20; B = exon 21–42; C = exon 43–50; D = exon 51+; P = point mutation; U = unknown

Cluster analysis of cardiac MR data did not reveal any specific clusters based on cardiac involvement patterns. The dendrogram is shown in (Fig. 4). A comparison between the groups defined by the deletion site revealed higher segmental ECV values in inferolateral and inferoseptal segments in the group with 51+ exon deletion compared to the other groups (*p* ≤ 0.05). This association did not change substantially after age adjustment. No other difference in the cardiac MRI data was observed (Table 5).

**Table 5:** Comparison of cardiac MR parameters in patients with different deletion sites in the dystrophin gene

Segment	Group A	Group B	Group C	Group D	*p*-value of ANOVA
Age [years]	12.3 ± 4.9	14.7 ± 8.4	14.5 ± 4.8	14.9 ± 2.6	0.72
LV EF [%]	64.9 ± 7.3	63.5 ± 10.7	58.3 ± 13.7	46.0 ± 16.6	0.092
LVMi [g/m^2^]	37.8 ± 4.8	37.2 ± 10.9	38.9 ± 15.2	46.9 ± 7.5	0.60
*LV EDVi [ml/m* ^*2*^ *]*	*52 (49–55)*	*56 (43–64)*	*48 (36–63)*	*61 (48–72)*	*0.87*
*LV ESVi [ml/m* ^*2*^ *]*	*18 (13–21)*	*21 (12–29)*	*16 (14–27)*	*35 (20–53)*	*0.42*
*LV SVi [ml/m* ^*2*^ *]*	*31 (30–38)*	*34 (30–35)*	*29 (25–35)*	*26 (17–28)*	*0.13*
Native T1 (anterior)	1029 ± 31	1082 ± 77	1067 ± 65	1045 ± 16	0.31
Native T1 (anteroseptal)	1029 ± 28	1052 ± 41	1032 ± 31	1036 ± 9	0.62
Native T1 (inferoseptal)	1029 ± 25	1054 ± 55	1033 ± 40	1059 ± 32	0.29
Native T1 (inferior)	1031 ± 32	1050 ± 44	1046 ± 58	1041 ± 35	0.88
Native T1 (inferolateral)	1040 ± 39	1037 ± 42	1057 ± 33	1058 ± 52	0.65
Native T1 (anterolateral)	1016 ± 38	1013 ± 36	1052 ± 68	1027 ± 43	0.38
Native T1 (global)	1029 ± 28	1049 ± 37	1047 ± 39	1045 ± 20	0.62
ECV (anterior)	0.262 ± 0.020	0.261 ± 0.013	0.269 ± 0.050	0.263 ± 0.039	0.97
ECV (anteroseptal)	0.255 ± 0.037	0.247 ± 0.019	0.259 ± 0.036	0.306 ± 0.103	0.28
ECV (inferoseptal)*	**0.248 ± 0.031**	**0.237 ± 0.009**	**0.252 ± 0.036**	**0.321 ± 0.066** ^**a.b**^	**0.010**
ECV (inferior)	0.250 ± 0.032	0.243 ± 0.008	0.265 ± 0.043	0.274 ± 0.031	0.53
ECV (inferolateral)*	**0.256 ± 0.049**	**0.256 ± 0.045**	**0.293 ± 0.067**	**0.391 ± 0.116** ^**a,b**^	**0.022**
ECV (anterolateral)	0.250 ± 0.040	0.253 ± 0.016	0.276 ± 0.048	0.344 ± 0.117	0.070
ECV (global)	0.253 ± 0.032	0.249 ± 0.008	0.269 ± 0.043	0.304 ± 0.047	0.17

Variables with the Gaussian distribution are expressed as the mean ± standard deviation; those with different distribution are in *italics* as the *median (lower quartile – upper quartile)*

Variables marked in **bold** and denoted with * have their ANOVA *p*-value < 0.05

Post hoc test results: a – group D vs. group A: *p* < 0.05; b – group D vs. group B: p < 0.05

*LV* left ventricle, *EF* ejection fraction, *LVMi* left ventricular mass index (g/m^2^), *EDVi* end-diastolic volume index, *ESVi* end-systolic volume index, *SVi* stroke volume index

There were neither significant intra- nor interobserver differences with the exception of native T1 in the anterior segment and ECV in the inferolateral and anterolateral segments: here, the lowest Kendall’s W was 0.85 in the case of intraobserver and 0.82 in the case of interobserver agreement, still corresponding with very good observer agreement.

## Discussion

To the best of our knowledge, this is so far the largest study using T1 mapping in DMD males and the first one assessing cardiac MR parameters in the Czech DMD/BMD population. The study highlights several important findings. It has demonstrated that regional and global native T1 relaxation time increased independent of the presence or absence of myocardial fibrosis. Patients in the late stages of cardiac involvement also had elevated ECV.

As expected, a significant part of the patients had decreased LV function (14%) and the percentage of patients with regional fibrosis detected by LGE was even higher (44%). While progressive worsening of LV function in this population is already well known and several publications describing frequent LGE have also been published [13–17], there are only very limited data concerning native T1 relaxation times in DMD/BMD patients. In our study, patients had higher myocardial T1 native relaxation time compared to control groups. This measurement was consistent in all 6 evaluated LV segments. There was no difference between patients with or without regional LGE. Therefore, DMD boys with normal LV function and no LGE had higher native T1 values than controls. These results confirm two earlier published studies. Soslow et al. [20] studied 31 DMD patients and also found significantly higher T1 times compared to controls (1045 vs 988 ms), independent of LVEF and LGE. Olivieri et al. [22] used MOLLI and SASHA (saturation recovery single shot acquisition) techniques in 20 DMD boys and described significantly higher T1 native times (using both techniques) in all segments.

These concordant results could mean that native T1 relaxation time can be a potential robust marker of very early cardiac involvement, earlier than any other imaging marker like LV functional decrease or regional myocardial fibrosis. These finding are in concordance with trails assessing T1 relaxation time in patients with non-ischemic cardiomyopathy. In several papers, native T1 correlated with LV dilatation and LV functional decline and was shown to be predictive of heart failure events and all-cause mortality [28–32].

Native T1 relaxation time could also become a novel parameter for the optimal start and evaluation of cardiac therapy. An earlier start of therapy could have important prognostic consequences as cardiomyopathy is one of the main causes of death in DMD patients [33]. Nevertheless, more research is needed.

In contrast to native T1 relaxation time, we have found increased ECV only in DMD patients with LGE. ECV of patients without LGE did not differ from the controls. These findings are slightly contradictory with some other works. Soslow et al. [20] found not only increased ECV in all DMD patients (0.31 vs. 0.24), but also in patients with normal LVEF (0.28) and negative LGE (0.29). Correspondingly, Starc et al. [21] showed significantly higher ECV in DMD group (0.29 vs. 0.24) with no significant difference between patients with and without LGE (0.30 vs. 0.27), where no LGE groups differed significantly from controls.

On the other hand, other studies reached similar results to our study. Florian et al. [19] also found increased ECV (0.29 vs. 0.24) in a BMD patients’ group, but this increase was exclusively in patients with cardiac involvement. Patients with normal LV function and with no LGE had the same ECV as the controls. Likewise, Olivieri et al. [22] described higher ECV in a DMD group without the possibility of distinguishing between the controls and DMD patients without fibrosis. These discrepancies could have several reasons. Most probably they reflect a combination of several factors, including low numbers of patients in the compared groups, generally very slight differences in ECV values and also difficulties in LGE assessment. LGE is commonly visually evaluated on the assumption it is a binary variable, but the myocardial fibrosis that is linked to the LGE is a continuous process, so that it is very likely that there are numbers of borderline LGE findings, where inclusion into the correct group is very subjective.

This is to authors’ knowledge first study to compare cardiac involvement assesses by MR and genetic background. Despite the progress on prediction of DMD/BMD phenotype, more detailed predictions are complicated and unreliable. It is almost impossible to predict the age until an out-patient care will remain possible and similarly it is very difficult to trace age dependency in the cardiac involvement. Nevertheless mutation position in higher exons (51st exon and higher) was found to be associated with mores severe ECV values in segments, previously described as typical for initial fibrosis identification [32]. These results suggest that in this patient group the cardiology care should be extremely cautious and aimed on the specific segment fibrosis assessment as early as reasonably possible. This shall further support the early pharmacotherapy indication according to subclinical MR findings even before manifested left ventricular dysfunction [33].

The study has several limitations. Some of them are related to the general limitations of T1 mapping [34, 35]. A larger sample size would be advantageous. However, the study involves a rare disease and cardiac MR cannot generally be performed in very young boys as well as in advanced DMD, often ventilated patients, so the relatively small population is further reduced. Moreover, the control group was rather small and was not perfect match in some parameters (age, weight, height). Finding a matched cohort for DMD patients is a highly complex matter. DMD boys with progressive skeletal muscle dystrophy frequently have smaller body than their corresponding peers [36]. Differences in basic characteristics were partially addressed by the use of multivariable models, where adjustment for age did not significantly change the results. Other groups also had to solve similar problems, mostly by not fully matching controls or by using previously published normal values [20, 37]. Besides, as it is unethical to use a contrast agent in healthy children and there is no large pool of such young boys without clear cardiac disease and indication for cardiac MR.

## Conclusion

The study demonstrated that cardiac mapping is a feasible, non-invasive method and provides a powerful incremental diagnostic value in the cohort of DMD patients. Regional and global native T1 relaxation time was increased independently of the presence or absence of myocardial fibrosis. Patients in late stages of DMD also had elevated ECV. Native T1 relaxation time seems to be a potential novel robust marker of very early cardiac involvement. Cardiac MR may provide clinically useful information even without contrast media administration.

## Abbreviations

ANCOVA: Analysis of covariance
ANOVA: Analysis of variance
BMI: Body mass index.
b-TFE: Balanced turbo-field echo
cm: Centimetre
DGC: Dystrophin-glycoprotein complex
DMD/BMD: Duchenne and Becker muscular dystrophy
ECV: Extracellular volume
EDV: End-diastolic volume
EDVi: End-diastolic volume index
EF: Ejection fraction
EF: Ejection fraction
ESV: End-systolic volume
ESV: End-systolicc volume
ESVi: End-systolic volume index
g: Gram
IR-TFE: Inversion-recovery turbo field echo
kg: Kilogram
LGE: Late gadolinium enhancement
LGE-: Negative late gadolinium enhancement
LGE +: Positive late gadolinium enhancement
LV: Left ventricle
LVMi: Left ventricular mass index
LVOT: Left ventricular outflow track
ml: Millilitre
mmol: Millimol
MOLLI: Modified Look-Locker inversion recovery
MR: Magnetic resonance
ms: Millisecond
PCR: Polymerase Chain Reaction
ROC: Receiver operating characteristics
SASHA: Saturation Recovery Single Shot Sequence
SAX: Short axis
SCMR: Society for Cardiovascular Magnetic Resonance
SSFP: Steady state free precession
SVi: Indexed stroke volume
T1: T1 relaxation time
TE: Echo time
TI: Inversion time
TR: Repetition time
WM: Wall motion
